# First Report of *mcr-10* in a Seafood-Borne ESBL-Producing *Enterobacter xiangfangensis* Strain

**DOI:** 10.1007/s00284-025-04179-0

**Published:** 2025-03-14

**Authors:** Christian Xedzro, Toshi Shimamoto, Liansheng Yu, Yo Sugawara, Motoyuki Sugai, Tadashi Shimamoto

**Affiliations:** 1 Laboratory of Food Microbiology and Hygiene, Graduate School of Integrated Sciences for Life, Hiroshima University, 1-4-4 Kagamiyama, Higashihiroshima, 739-8528 Japan; 2 Antimicrobial Resistance Research Center, National Institute of Infectious Disease, 4-2-1 Aoba-Cho, Higashimurayama, Tokyo, 189-0002 Japan

## Abstract

*mcr-10* is among the growing families of newly identified plasmid-mediated mobile colistin-resistance genes. In this study, we identified an *mcr-10* gene in a seafood-borne extended-spectrum *β*-lactamase (ESBL)-producing *Enterobacter xiangfangensis*. *E. xiangfangensis* strain, B12-S77, was subjected to whole genome sequencing using Illumina MiSeq and Oxford Nanopore Technologies. Bioinformatic analysis was performed using tools from the Center for Genomic Epidemiology. The minimum inhibitory concentration (MIC) of 19 antibiotics was determined by the broth microdilution method. Transferability of *mcr-10*-carrying plasmid was investigated by the conjugation experiment. The strain exhibited a multidrug-resistant (MDR) phenotype against more than three classes of antibiotics but remained susceptible to colistin and polymyxin B. *mcr-10* was identified on a fused conjugative plasmid of the IncFIB (K):FII (Yp) backbone adjacent to the XerC-type tyrosine recombinase-gene. At least one insertion sequence (IS) was identified in both the downstream and upstream regions of the *xerC-mcr-10* conserved region, indicating that this region may contribute to *mcr-10* mobilization or integration into the bacterial genome. The strain belonged to sequence type (ST) 143 and carried the *nlpI* and *mrkA* virulence genes, which promote fimbrial adhesion or biofilm formation in enteric bacteria. This report provides novel insights into the emergence of *mcr-10* in seafood-borne bacteria, and highlights the importance of surveillance in the seafood supply chain.

**Supplementary Information:**

The online version contains supplementary material available at 10.1007/s00284-025-04179-0.

## Introduction

The *mcr-10* was first identified in the IncFIA pMCR10_090065 from *Enterobacter roggenkampii* clinical strain recovered in 2016 at the West China Hospital [1]. *mcr-10* encodes an enzyme that confers resistance to colistin, a last-resort antibacterial medication with excellent bactericidal activity for the treatment of patients with severe multidrug-resistant (MDR) infections, particularly those caused by *Enterobacteriaceae*. Subsequently, it was identified in *Enterobacteriaceae* in various countries [2–4]. Notably, the co-occurrence of *mcr-10* and carbapenemase-encoding genes has been reported [4]. Reports also confirmed the co-existence of *mcr-9* and extended-spectrum β-lactamase (ESBL) genes in *Enterobacter* spp. [5], though not the co-occurrence of *mcr-10* and ESBL genes in seafood-borne *Enterobacteriaceae*. The co-harboring of *mcr-10* and ESBL genes may further jeopardize clinical anti-infective therapeutics. In recent years, the frequency of nosocomial infections caused by *Enterobacter* spp. has increased [6]. As colistin resistance and ESBL genes have evolved together, the emergence of high-risk strains is expected to become a serious public health threat.

Plasmids play a key role in the horizontal gene transfer of mobile genetic elements, facilitating their dissemination through conjugation. In some cases, nonconjugative plasmids can be transmitted via replicative transposition and recombination with conjugative helper plasmids, facilitating the distribution of resistance genes [7]. To date, the coexistence of *mcr-10* and ESBL genes has not been reported in seafood products. Additionally, no foodborne *mcr-10*-carrying *Enterobacteriaceae* have been reported in Japan, although two reports have identified this gene in *E. roggenkampii* isolated from dogs and clinical specimens [8, 9]. The epidemiology of *mcr*-related colistin resistance in Japan is primarily associated with *Escherichia coli* and *Enterobacter* spp. recovered from livestock or clinical specimens [9–12]. However, monitoring *mcr*-genes in seafood products could expand our understanding and provide valuable insights into the dissemination of these genes and their role in mediating polymyxin resistance. In a prospective surveillance program aimed at investigating the presence of the plasmid-mediated mobile colistin resistance (*mcr*) gene, we isolated an *mcr-10-*carrying bacterium producing an ESBL gene from a retail seafood sample in Hiroshima, Japan. Detailed genetic analysis and comparisons were performed to investigate the role of this strain in the dissemination of antibiotic resistance genes. To our knowledge, this is the first report of *mcr-10* co-existing with *bla*_SHV-12_ in a seafood-borne *E. xiangfangensis* strain. MDR phenotypes were observed in the parental strain and non-wild-type *Escherichia coli* transconjugants.

## Materials and Methods

### Bacterial Isolation and Antimicrobial Susceptibility Testing

Between December 2022 and August 2023, 50 unfrozen seafood samples were purchased from several retail shops in Hiroshima, Japan, using a simple random sampling process. Bacterial isolation was performed as previously described [13, 14]. Briefly, a 25 g portion of each sample was homogenized in 225 mL of buffered peptone water (Nissui Pharmaceutical Co., Ltd., Tokyo, Japan) containing 2% NaCl. The resulting homogenates were pre-enriched at 37 °C for 6‒8 h and then plated on MacConkey agar (Eiken Chemical Co., Ltd., Tochigi, Japan) containing streptomycin (50 µg/mL), colistin (2 µg/mL), ampicillin (100 µg/mL), or meropenem (2 µg/mL). Additionally, 1 mL of each pre-enriched culture was added to 9 mL of Luria–Bertani (LB) broth (Nacalai Tesque, Inc., Kyoto, Japan) with or without streptomycin and ampicillin and incubated at 37 °C for 24 h. Enriched streptomycin broth cultures were plated on MacConkey agar containing streptomycin (50 μg/mL), while the enriched ampicillin-containing cultures were plated on MacConkey agar containing ampicillin (100 µg/mL) or meropenem (2 μg/mL). Broth cultures with colistin were not prepared (to avoid inducible colistin resistance); instead, antibiotic-free LB broth suspensions were incubated overnight, then plated on MacConkey agar containing colistin (2 μg/mL). These antibiotics were used not only to provide selective pressure and enhance selectivity of a wide range of resistant bacterial strains but also to stimulate or reflect the real-world conditions under which multidrug-resistant strains are most likely to thrive, which is a key in studies related to resistance mechanisms. The plates were incubated, and several morphologically distinct colonies per plate were selected for analysis. A total of 412 Gram-negative bacteria were recovered and purified on LB agar supplemented with 2% NaCl. These isolates were screened using PCR to detect families of the plasmid-mediated mobile colistin resistance genes, *mcr-1‒10*, as previously described with minor changes [15, 16]. While none of the isolates detected positive for an *mcr* gene, one strain, B12-S377, which was isolated in July 2023 from a fish (Japanese pilchard, *Sardinops melanostictus*) sample detected positive for *mcr-10*. The fish sample was confirmed to have originated from Japan. The MICs of ampicillin, cefotaxime, ceftazidime, ceftriaxone, cefoperazone, cefoxitin, aztreonam, meropenem, kanamycin, gentamicin, streptomycin, chloramphenicol, ciprofloxacin, norfloxacin, tetracycline, trimethoprim, fosfomycin, colistin, and polymyxin B were determined using the broth microdilution method of Clinical Laboratory Standard Institute (CLSI, 2020) [17]. For colistin and polymyxin B, the EUCAST breakpoints were employed (https://www.eucast.org/).

### Whole Genome Sequencing and Assembly

To understand the genetic profile of the strain, total genomic DNA was extracted using the Qiagen Genomic-tip 20/G kit (Qiagen, Hilden, Germany), and the complete genome was sequenced using Illumina MiSeq Reagent Kit v3 and Oxford Nanopore Technologies. A de novo assembly was performed using Unicycler v0.4.8 (https://github.com/rrwick/Unicycler).

### Sequence Annotation, Analysis, and Comparison

Generated contigs were annotated using RAST*tk* (https://rast.nmpdr.org/rast.cgi) and DFAST bioinformatic tool (https://dfast.ddbj.nig.ac.jp/). The downstream genome analysis was performed on the Center for Genomic Epidemiology website (http://www.genomicepidemiology.org/services/). A pairwise average nucleotide identity (ANI) analysis was performed using JSpeciesWS (https://jspecies.ribohost.com/jspeciesws/) to identify the specific species of *Enterobacter* for strain B12-S377. A > 95% ANI value was used as the cut-off to type bacterial species. An in silico genome-genome comparison based on digital DNA-DNA hybridization (dDDH) was performed using the Type (Strain) Genome Sever (TYGS) (https://tygs.dsmz.de/user_requests/new). BLAST Ring Image Generator (https://sourceforge.net/projects/brig/) was used to produce a circular map to compare the plasmid identified in this study with similar plasmids in the NCBI database. The genetic context surrounding the *mcr-10* was visualized with Easyfig v2.2.5 (https://mjsull.github.io/Easyfig/). The transferability of *mcr-10*-carrying plasmid was investigated using a filter-mating conjugation assay [18].

### Biofilm Assay

The crystal violet staining method was employed to assess the biofilm-producing capacity of the strain [19]. The strain was cultured overnight and subsequently diluted (1:100) in LB medium. Then 200 μL of the suspension was transferred into V-bottom 96-well microtiter plate (Micro test plate 96-well; Nerbe Plus, Germany). Following incubation at 37 °C for 24 h, the planktonic cells were carefully removed, and the wells were washed three times with distilled water. The adhering biofilms in the wells were stained with crystal violet (0.1%, w/v) for 15 min, and washed with distilled water (three times). The stained biofilms were solubilized using 95% ethanol (v/v). The relative biofilms were quantified by measuring the optical density (OD) at 570 nm using a microplate reader (Multiskan Sky; Thermo Scientific, Finland). LB broth without inoculation served as a negative control. In order to assess whether the biofilm formation of the strain is affected by the presence of *nlpI* and *mrkA* virulence genes, we included *E. cloacae* CST17-2 [17]—a strain from the same genus as our test strain but lacking these virulence genes—to monitor any differences in biofilm formation. *E. cloacae* CST17-2 was chosen for comparison due lack of a control *E. xiangfangensis* strain.

### Nucleotide Sequence Accession Numbers

The complete genome sequence of *E. xiangfangensis* B12-S377 has been deposited in DDBJ/ENA/GenBank under the BioProject and BioSample accession numbers PRJDB18940 and SAMD00824839, respectively.

## Results and Discussion

The strain B12-S377 shares 99.87% ANI value with the reference strain *E. hormaechei* subsp. *xiangfangensis* (accession number CP017183). In the dDDH analysis, it exhibited 96.1% similarity with same type strain *E. xiangfangensis.* The ANI and dDDH values exceeded the proposed thresholds (ANI, > 95 to 96%; dDDH, > 70%) for defining bacterial species [20]. A report by Wu et al. [21] has shown that *E. hormaechei* subsp. *xiangfangensis* is not a subspecies of *E. hormaechei* but rather belongs to the species *E. xiangfangensis*, indicating that B12-S377 is indeed *E. xiangfangensis*. The strain was highly resistant to a wide range of antibiotics, including third-generation cephalosporins, but remained susceptible to colistin and polymyxin B upon MIC determination (Table 1). It is important to note that though *mcr-10* has been associated with colistin resistance or reduced susceptibility to colistin in previous studies [22], it may not be actively expressed at high levels in certain strain background. This can lead to insufficient production of *mcr-10* protein, which plays a key role in modifying lipid A and conferring resistance. Furthermore, the occurrence of colistin susceptibility in *mcr-10*-carrying bacterium has been previously reported [23]. Therefore, some *mcr* variants are incapable of conferring colistin resistance due to various factors, such as reduced gene expression, lower plasmid copy number, and increased fitness cost [23, 24]. Additionally, the presence of *mcr-10* does not consistently correlate with elevated colistin MIC, as various factors, including regulatory elements and gene expression levels, influence colistin susceptibility [3]. It is also conceivable that large naturally occurring plasmids may affect *mcr* expression [1], particularly since our *mcr-10* was found on a 136 976 bp plasmid. The expression of *mcr-10* can vary based on the genetic context, including surrounding regulatory parameters and plasmid characteristics, which can greatly impact the mechanisms of colistin resistance [25]. The identification of a colistin-susceptible phenotype in this study, despite the presence of *mcr-10* in the strain, is not surprising, as epidemiological investigations and susceptibility testing continue to uncover diverse colistin resistance phenotypes among different *mcr*-carrying bacteria [18].

**Table 1:** MIC of antimicrobials for *mcr-10*-carrying *E. xiangfangensis* strain, B12-S377, and its *E. coli* transconjugant

Isolate	MIC (µg/mL)																		
	AMP	CTX	CAZ	CRO	CFP	FOX	ATM	MEM	KAN	GEN	STR	CHL	CIP	NOR	TET	TMP	FOF	CST	PMB
B12-S377	** > 512**	** > 32**	** > 128**	** > 32**	** > 256**	** > 128**	**128**	< 0.06	0.25	0.5	**128**	8	**2**	2	**64**	** > 128**	** > 1024**	0.5	0.5
TC1	** > 512**	** > 32**	** > 128**	** > 32**	** > 256**	**128**	**64**	< 0.06	0.25	0.5	**64**	ND	**0.5**	2	**32**	** > 128**	ND	0.5	0.5
TC2	** > 512**	** > 32**	** > 128**	** > 32**	** > 256**	** > 128**	**64**	< 0.06	0.5	1	**64**	8	**1**	1	**32**	** > 128**	ND	0.5	0.5
*E. coli* J53	16	0.13	0.5	0.25	1	4	0.5	< 0.06	0.5	2	4	2	0.06	< 0.13	2	2	ND	ND	ND
*E. coli* ATCC 25922	8	0.25	0.25	0.5	0.13	4	0.13	< 0.06	4	4	4	2	0.13	0.06	2	0.5	ND	0.13	0.5

Bold values indicate the resistance

*TC1/TC2* transconjugants, *AMP* ampicillin, *CTX* cefotaxime, *CAZ* ceftazidime, *CRO* ceftriaxone, *CFP* cefoperazone, *FOX* cefoxitin, *ATM* aztreonam, *MEM* meropenem, *KAN* kanamycin, *GEN* gentamicin, *STR* streptomycin, *CHL* chloramphenicol, *CIP* ciprofloxacin, *NOR* norfloxacin, *TET* tetracycline, *TMP* trimethoprim, *FOF* fosfomycin, *CST* colistin, *PMB* polymyxin B

Multilocus sequence typing revealed that it belonged to sequence type (ST) 143 with the following allelic profile: *dnaA*_10-*fusA*_21-*gryB*_9-*leuS*_44-*pryG*_45-*rplB*_4-*rpoB*_40. Whole-genome analysis showed that strain B12-S377 contained a 4 772 906 bp chromosome and two plasmids, pS377_mcr-10 and pS377_A, with sizes of 136 976, and 2 493 bp, respectively (Table 2). The chromosome harbors *bla*_ACT-25_ and *fosA* resistance genes conferring cephalosporin and fosfomycin resistance, respectively. Notably, we detected OqxAB-resistance nodulation division efflux pump systems encoding *oqxA* and *oqxB* (Table 2), which mediate resistance to multiple antimicrobial agents, including fluroquinolones [26]. Additionally, the chromosome contained a multidrug efflux pump system (EmrAB/D-OM) and eight ISs. *mcr-10* was located on a fused plasmid of the IncFIB(K):FII (Yp) backbone and named pS377_mcr-10. The plasmid carried 11 ISs and MDR genes, including *bla*_SHV-12_, *bla*_TEM-1_, *sul2*, *aph(3'')-Ib*, *aph(6)-Id*, *qnrB1*, *dfrA14*, and *tet*(A) (Table 2). Besides, the strain carried two virulence genes, *nlpI* and *mrkA*, which promote fimbrial adhesion and/or biofilm formation in enteric bacteria [27, 28]. Although the overall biofilm quantity was low, the strain produced significantly higher amount of biofilm (Mann Whitney *U* test; *P* = 2.138E-8) compared to *E. cloacae* CST17-2 (Fig. S1), suggesting that the virulence genes may have contributed to the strain’s biofilm formation. Full sequence query of pS377_mcr-10 against NCBI database revealed that it has high degree of genetic identity with pK528_mcr-10 (accession number CP095178, 99.95% nucleotide identity at 99% query coverage) in *E. hormaechei* isolated from a healthy dog [22], and was also similar to a portion of pK475-2_mcr-10 (accession number CP095167, 99.95% nucleotide identity at 61% query coverage) in *E. roggenkampii* isolated from a healthy cat [22] in China. Downstream analysis of the resistome indicated that pS377_mcr-10 shared multiple antibiotic resistance genes, similar to pK528_mcr-10 [22], suggesting that the two plasmids might have evolved from a common ancestor.

**Table 2:** Genomic features of B12-S377

Genome	Size (bp)	GC content (%)	Antimicrobial resistance genes	Virulence genes
Chromosome	4 772 906	55	*bla*_ACT-25_, *fosA*, *oqxA*, *oqxB*	*nlpI*
pS377_mcr-10	136 976	52	*sul2*, *aph(3'')-Ib*, *aph(6)-Id*, *bla*_TEM-1_, *bla*_SHV-12_, *qnrB1*, *dfrA14*, *mcr-10.1*, *tet*(A)	*mrkA*
pS377_A	2 493	52	–	–

**Fig. 1:**
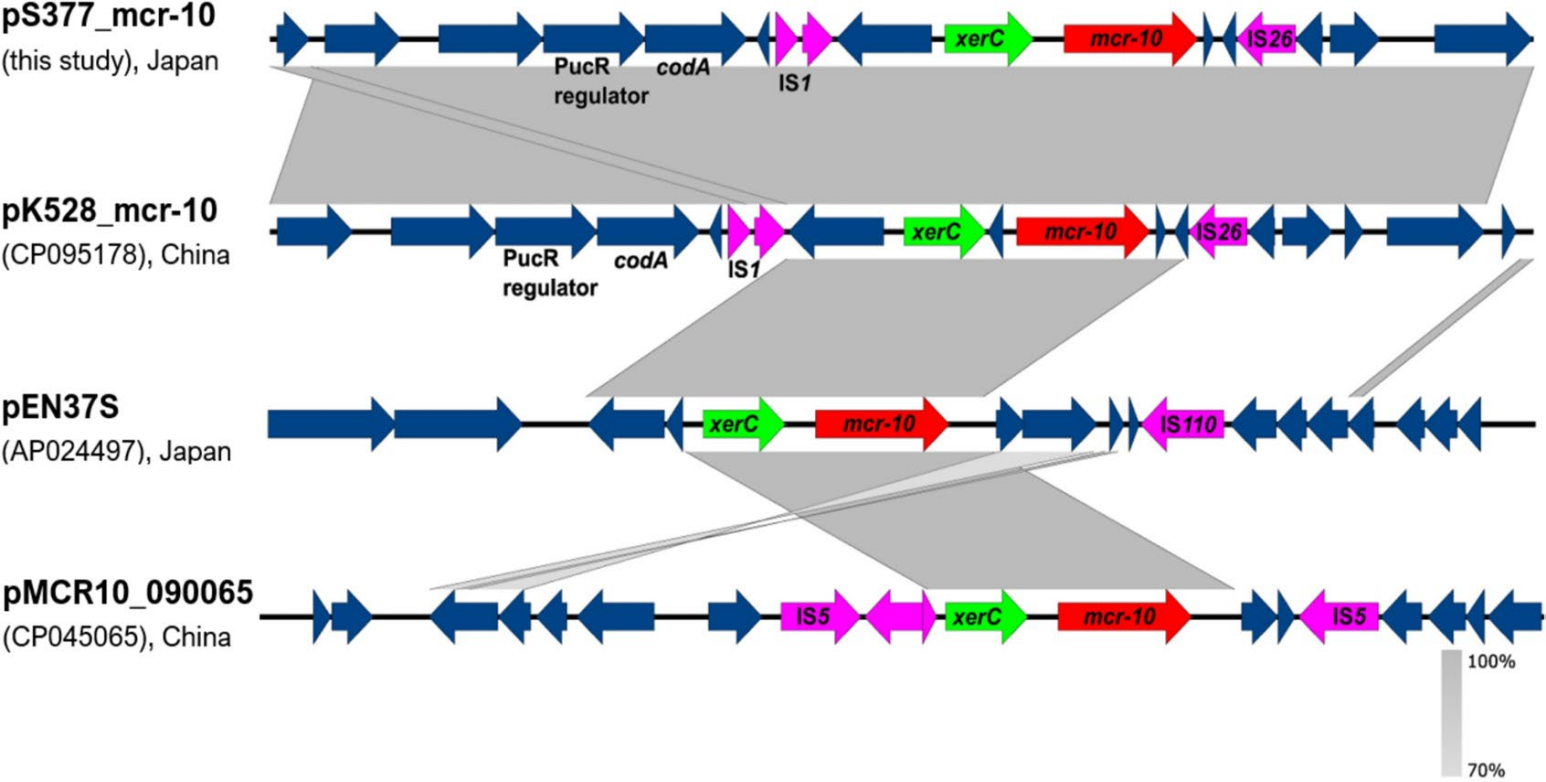
Colinear analysis and comparison of the genetic context of *mcr-10* with homologous regions of other *mcr-10*-harboring plasmids retrieved from the NCBI database. Homologous regions with significant nucleotide identities are shown by shading. The *mcr-10*, *xerC*, and insertion sequences (ISs) are indicated by red, green, and purple colors, respectively. The figure was generated using Easyfig tool and manually annotated after BLASTn analysis of those regions

In pS377_mcr-10, *mcr-10* was located immediately downstream of an XerC-type tyrosine recombinase encoding *xerC* (Fig. 1). The *xerC-mcr-10* is highly conserved in *mcr-10*-carrying *Enterobacteriaceae* [1–4]. A previous report showed that XerC-type tyrosine recombinase is associated with site-specific recombination mechanisms, leading to the integration or mobilization of *bla*_NMC-A_ and *bla*_IMI_ carbapenemase genes in some *Enterobacter* spp. [29]. Therefore, *mcr-10* mobilization may be mediated by this specific *xerC*-type tyrosine recombination system, although plasmids may also play a key role in the spread of *mcr-10*. Various ISs were found in the upstream and/or downstream regions of *mcr-10*, but the genetic contexts were different. Insertion sequences are the predominant genetic elements that contribute to the remodeling and spread of resistance determinants in Gram-negative bacteria.

**Fig. 2:**
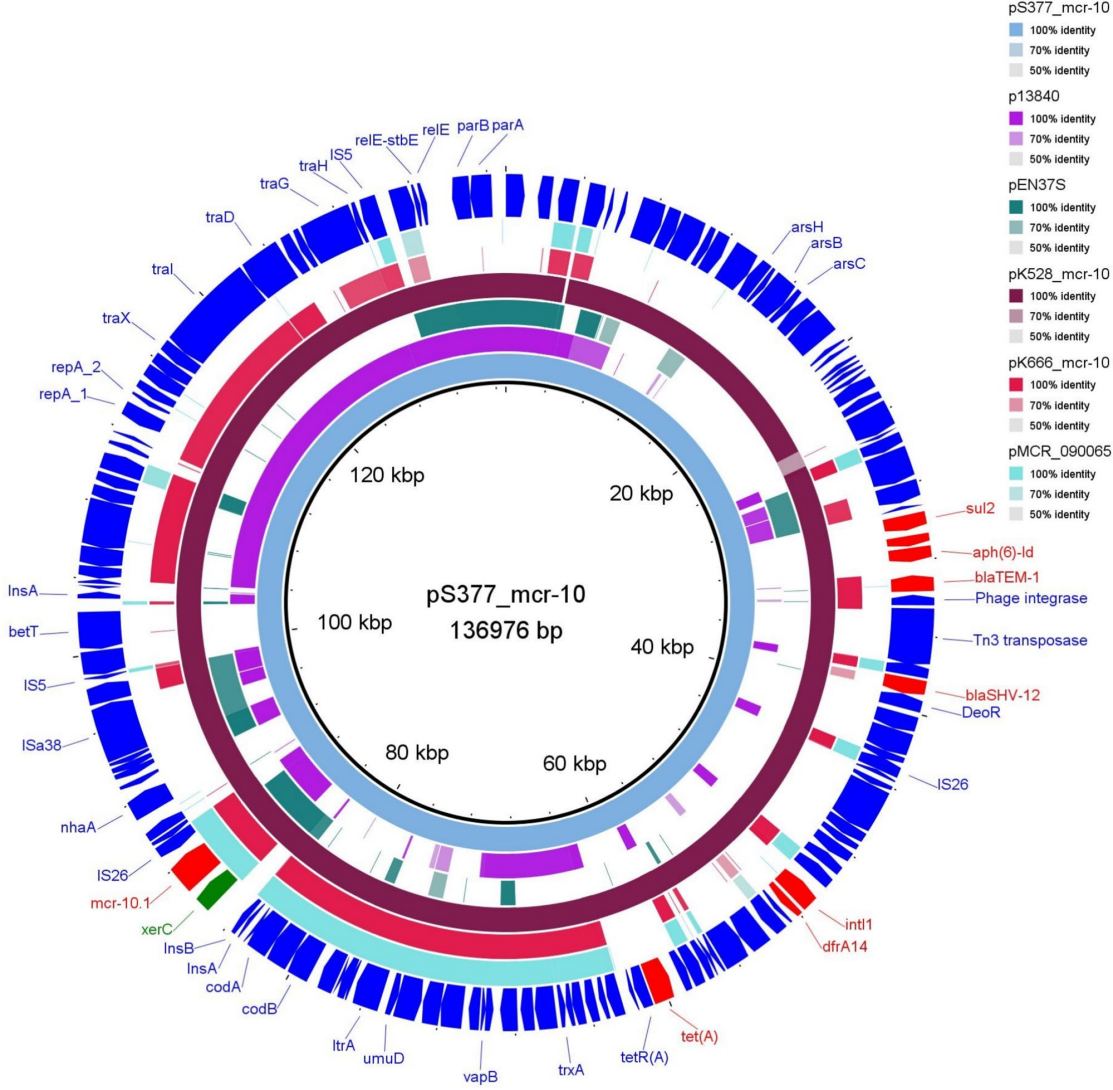
Circular comparison of pS377_mcr-10 (reference, this study) against *mcr-10*-bearing IncF plasmids. The plasmids were added in the following order from the inner ring: pS377_mcr-10 (this study), p13840 (CP083820), pEN37S (AP024497), pK528_mcr-10 (CP095178), pK666_mcr-10 (CP095171), and pMCR10_090065 (CP045065). The outer ring represents genes and open reading frames of the reference plasmid, with arrow heads indicating the transcriptional orientations. The gaps indicate low or no similarities among the plasmid sequences. Some genes and insertion sequences were hidden from the outer ring. Sequence similarities are shown using BLAST Ring Image Generator

Furthermore, IS*1* and IS*26* were located in the upstream and downstream regions, respectively, of *mcr-10* in pS377_mcr-10, sharing a genetic arrangement similar to that of pK475-2_mcr-10 (Figs. 1, 2). These genetic contexts differed from pMCR10_090065 (accession number CP045065), where *mcr-10* was first identified, in which a composite transposon was formed by two copies of truncated IS*903B* flanking *mcr-10*. The genetic context of the ESBL-encoding gene, *bla*_SHV-12_, carried by a Tn*3*-type family transposase (not shown), was examined. For *bla*_SHV-12_ flanking regions, a hypothetical protein and putative *deoR* transcriptional regulator were located 53 bp upstream and 782 bp downstream, respectively. This regulator plays a role in many physiological processes in bacteria, including nucleotide metabolism, virulence, and metal resistance [30]. To explore the transconjugation mechanism of *mcr-10*, we analyzed the genetic context of the gene and plasmid. Notably, the genes encoding the conjugative transfer relaxase, TraI-TraX, and type IV conjugative transfer system coupling protein, TraD-TraG, were located far downstream of *mcr-10*. Filter-mating conjugation was performed at 37 °C using azide-resistant *E. coli* J53 as the recipient and B12-S377 as the donor. Transconjugants were selected on LB agar containing sodium azide and ampicillin (100 µg/mL each agent). Interestingly, pS377_mcr-10 was successfully transferred to the recipient cells. MIC determinations showed that the transconjugants exhibited antibiotic resistance patterns similar to those of the parental strain *E. xiangfangensis* (Table 1).

## Conclusion

Our results suggest that *mcr-10*-positive plasmids may silently disseminate through cross-sector transmission in aquatic environments, humans, and animals. This MDR strain is expected to have spread throughout the seafood supply chain in Japan. Considering that seafood is often served raw in Japanese cuisine, the discovery of such a highly resistant strain presents a consumer food safety concern and may compromise the patronage of seafood products. Although *mcr-10* did not mediate colistin resistance, this study presents emerging insights into mobile *mcr-10* in Japan, with the first report of *mcr-10* gene in seafood worldwide (to the best of our knowledge). The mobility of the associated plasmid indicates a high chance of colistin resistance transmission within and between bacterial species, as the *mcr-10* gene has the potential to be expressed in other strain background. Since colistin is a last-resort antibiotic for treating patients with severe MDR infections, robust policies are needed to enhance antimicrobial stewardship and curb the spread of *mcr* genes and host microbiomes. Additionally, the coexistence of *mcr-10* and ESBL genes may complicate clinical treatments.

Our findings are essential for effective countermeasures, including surveillance, and provide baseline information for further research in the seafood industry.

## Supplementary Information

Below is the link to the electronic supplementary material.

Supplementary file1 (DOCX 117 KB)

## Data Availability

All data generated in this study are included in the article. Raw data images of PCR bands will be provided upon request.
