# Supplementary material for: First Report of mcr-10 in a Seafood-Borne ESBL-Producing Enterobacter xiangfangensis Strain
Source: Curr Microbiol. 2025 Mar 14;82(5):194. doi: 10.1007/s00284-025-04179-0 (PMC11909031; doi:10.1007/s00284-025-04179-0)
Supplement: Supplementary file 1 — Supplementary file1 (DOCX 117 KB) [file 284_2025_4179_MOESM1_ESM.docx]

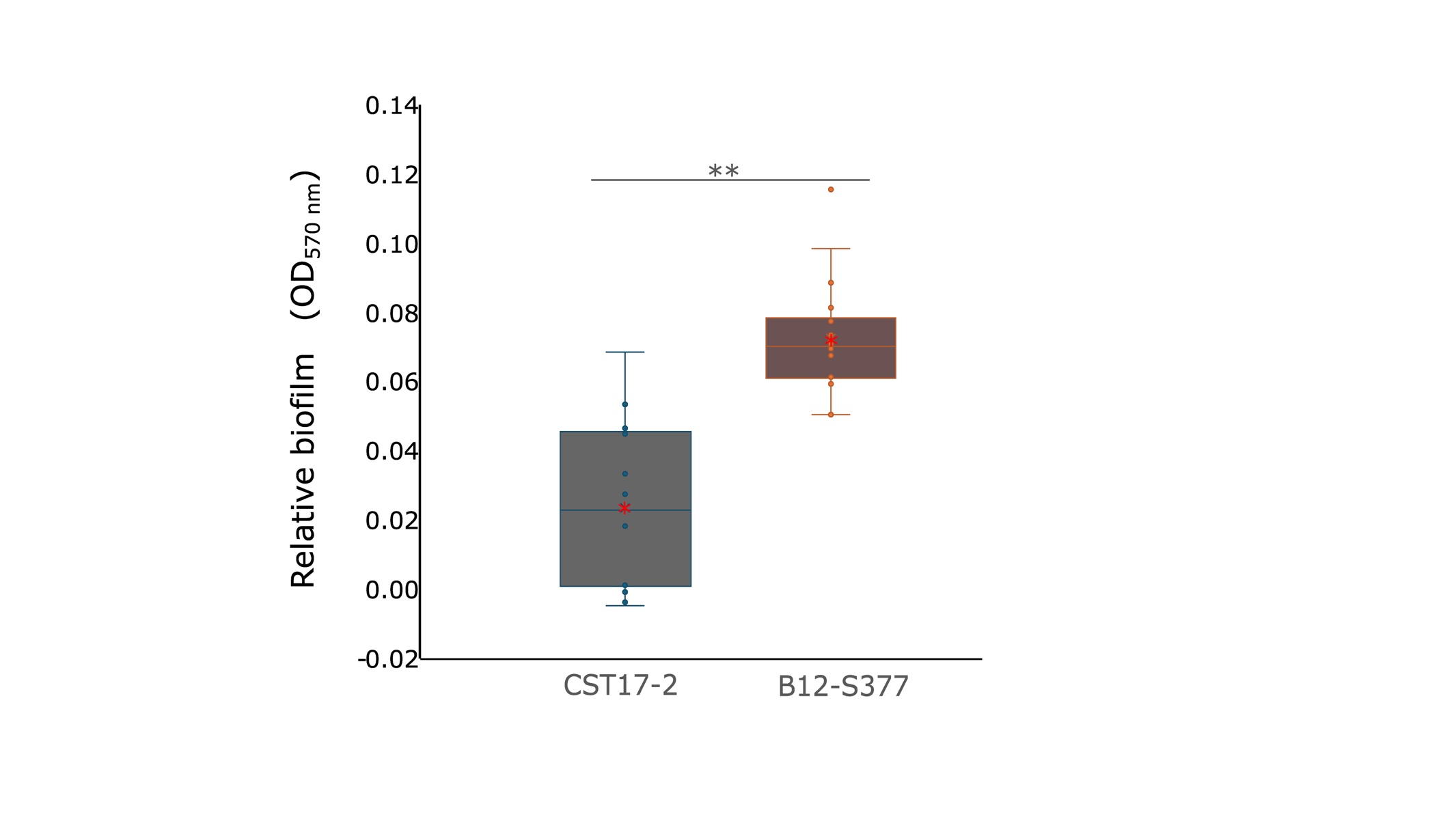


**Fig. S1** Statistical box plots representing the biofilm mass of *E. xiangfangensis* B12-S77 in comparison to *E. cloacae* CST17-2. Data means are indicated by red asterisks. The double asterisks denote significance as determined by the Mann Whitney U Test (*p*=2.138E-8).
